# Supplementary material for: Radiomics of MRI for the Prediction of the Pathological Response to Neoadjuvant Chemotherapy in Breast Cancer Patients: A Single Referral Centre Analysis
Source: Cancers (Basel). 2021 Aug 25;13(17):4271. doi: 10.3390/cancers13174271 (PMC8428336; doi:10.3390/cancers13174271)
Supplement: Supplementary file 1 [file cancers-13-04271-s001.zip › cancers-1359842-supplementary.pdf]

Article

Radiomics of MRI for prediction of pathological response to neoadjuvant chemotherapy in breast cancer: a single referral centre analysis.

Filippo Pesapane, Anna Rotili, Francesca Botta, Sara Raimondi, Linda Bianchini, Federica Corso, Federica Ferrari, Silvia Penco, Luca Nicosia, Anna Bozzini, Maria Pizzamiglio, Daniela Origgi, Marta Cremonesi and Enrico Cassano

Supplementary materials

Table S1. Our standardized breast MRI acquisition protocol. TSE: turbo spin echo, TE: echo time, FA: flip angle, FOV: field of view, mm: millimeters, TR: repetition time, ms: milliseconds.

	**1.5 T MRI scanner (Optima MR450W, General Electric Healthcare) with a dedicated 8 channels breast coil**
**T2-weighted imaging**	Axial fat-saturated T2-weighted imaging
	spatial resolution of 3.6 × 2.7 × 3.5 mm
	TR 3914 ms, TE 70-111 ms, FA 150°, FOV 240 × 100 mm, base resolution 256 with voxel 1.1 × 0.9 × 3.0 mm, number of averages 1
**Diffusion weighted imaging**	Transverse REVEAL, 3 mm slice thickness
	b-values 0, 800 (calculated)
	TR 5100 ms, TE 90 ms, number of averages: 2
**Dynamic contrast enhanced imaging**	Axial fat-saturated T1-weighted imaging
	1.4 mm slice thickness, , slice spacing 0.7 mm, field of view 350 × 350 mm^2
	Perfusion temporal resolution : 15 sec (total time lenght: 360 sec)<br>TR 7.39 ms, TE 3.44 ms, FA 14

Table S2. Textural features and features extraction details.

**Textural features**	which identify specific patterns in the intensity distribution, were calculated from specific matrixes, namely: Gray Level Co-occurrence Matrix (GLCM), Gray Level Run Length Matrix (GLRLM), Gray Level Size Zone Matrix (GLSZM), Neighbouring Gray Tone Difference Matrix (NGTDM), and Gray Level Dependence Matrix (GLDM).
**Features extraction**	Extraction was performed on both original and filtered images, considering the following filters available in PyRadiomics: Laplacian of Gaussian - LoG -, Wavelet, Square, Square Root, Logarithm, Exponential, and Local Binary Pattern 2D - LBP2D -. A 6.0 mm sigma in the LoG filter and one level for the wavelet decompositions were selected. PyRadiomics normalization was applied by setting the images mean signal intensity to 300 and standard deviation (SD) to 100. The image intensities were discretized with a fixed bin-width set to 5.

Table S3. Statistical analysis of radiomics workflow. *FDR: False Discovery Rate, RF: Random Forests.*

**Radiomic analysis**	we performed a univariate analysis for the association between each radiomic feature with pCR to NACT by using the non-parametric Wilcoxon-Rank-Sum test with FDR correction. Then, we applied a two-steps procedure, namely features reduction and model construction.
**Feature reduction**	• firstly we removed from the dataset the radiomic features with near zero variance and with near one correlation (Spearman coefficient>0.99) with other features. Specifically, if two variables have a high correlation, the function looks at the mean absolute correlation of each variable and removes the variable with the largest mean absolute correlation.<br>• secondly, an in-house function was implemented to identify clusters of radiomic features with Spearman's coefficient ≥0.75 (supplementary table 1). We then selected only one feature for each cluster (i.e., the most associated with the outcome based on Wilcoxon Rank Sum test) and discharged the others
**Model construction**	we applied different machine learning methods for classification, including penalized regression with optimization of alpha and lambda hyperparametrs, K-Nearest Neighbour, Support Vector Machine and Random Forest (RF). We here present the approach for which a better classification was obtained. Thus, we implemented RF with an internal repeated-Cross Validation (CV, 5 × 10 fold) on the entire dataset using standardized radiomic features and adjusting for imbalanced outcomes with SMOTE sampling (17). The *caret* package with method "ranger" in R4.0-software was used for RF construction (18). We extracted the predictor importance (Gini index) of the top ten radiomic features and obtained a radiomic score as the prediction probabilities averaged on the 5 × 10 RF.

Table S4. Clinical and biological variables of the study population included in multivariable association analysis with response to therapy.

Characteristics	OR* (95%CI)
**Age^**	0.98 (0.92–1.05)
**Molecular subtype**	
Luminal A	1.00 (Reference)
Luminal B	1.18 (0.17-8.24)
Her2-positive	**24.78 (4.51-136.21)**
Basal-like	**8.63 (1.86-40.02)**
**Ki67^**	
≥ 20%	1.00 (Reference)
< 20%	3.53 (0.31–40.95)

CI = confidence Interval; OR = odds ratio; *multivariable logistic regression model. The following variables: ER, PR, HER2 were not included because they are used to define molecular subtype; NACT therapy was not included because it is dependent by molecular subtype.

Table S5. Radiomic features. List of radiomic features shown for category, extracted with the package PyRadiomics from the original images. The Shape parameters are calculated on the original (not filtered) images only. All the other features are calculated both on the original images and for each filter applied (7 filters), for a total of 1037 features. The mathematical definition of each feature reported in this Table is available at https://pyradiomics.readthedocs.io/en/latest/features.html.

**Shape**	**First Order**	**GLCM**	**GLRLM**
Elongation	10Percentile	Autocorrelation	GrayLevelNonUniformity
Flatness	90Percentile	ClusterProminence	GrayLevelNonUniformityNormalized
LeastAxisLength	Energy	ClusterShade	GrayLevelVariance
MajorAxisLength	Entropy	ClusterTendency	HighGrayLevelRunEmphasis
Maximum2DDiameterColumn	InterquartileRange	Contrast	LongRunEmphasis
Maximum2DDiameterRow	Kurtosis	Correlation	LongRunHighGrayLevelEmphasis
Maximum2DDiameterSlice	Maximum	DifferenceAverage	LongRunLowGrayLevelEmphasis
Maximum3DDiameter	MeanAbsoluteDeviation	DifferenceEntropy	LowGrayLevelRunEmphasis
MeshVolume	Mean	DifferenceVariance	RunEntropy
MinorAxisLength	Median	Id	RunLengthNonUniformity
Sphericity	Minimum	Idm	RunLengthNonUniformityNormalized
SurfaceArea	Range	Idmn	RunPercentage
SurfaceVolumeRatio	RobustMeanAbsoluteDeviation	Idn	RunVariance
VoxelVolume	RootMeanSquared	Imc1	ShortRunEmphasis
	Skewness	Imc2	ShortRunHighGrayLevelEmphasis
	TotalEnergy	InverseVariance	ShortRunLowGrayLevelEmphasis
	Uniformity	JointAverage	
	Variance	JointEnergy	
		JointEntropy	
		MCC	
		MaximumProbability	
		SumAverage	
		SumEntropy	
		SumSquares	

**GLSZM**	**NGTDM**	**GLDM**
GrayLevelNonUniformity	Busyness	DependenceEntropy
GrayLevelNonUniformityNormalized	Coarseness	DependenceNonUniformity
GrayLevelVariance	Complexity	DependenceNonUniformityNormalized
HighGrayLevelZoneEmphasis	Contrast	DependenceVariance
LargeAreaEmphasis	Strength	GrayLevelNonUniformity
LargeAreaHighGrayLevelEmphasis		GrayLevelVariance
LargeAreaLowGrayLevelEmphasis		HighGrayLevelEmphasis
LowGrayLevelZoneEmphasis		LargeDependenceEmphasis
SizeZoneNonUniformity		LargeDependenceHighGrayLevelEmphasis
SizeZoneNonUniformityNormalized		LargeDependenceLowGrayLevelEmphasis
SmallAreaEmphasis		LowGrayLevelEmphasis
SmallAreaHighGrayLevelEmphasis		SmallDependenceEmphasis
SmallAreaLowGrayLevelEmphasis		SmallDependenceHighGrayLevelEmphasis
ZoneEntropy		SmallDependenceLowGrayLevelEmphasis
ZonePercentage		
ZoneVariance		

Table S6. In-house function used to perform cluster analysis (R software).

# **function for creating a matrix for each cluster**	# **loop for cluster choice based on minimum correlation**
x <- function(dati, tree, n_cl)<br>{step1 <- cbind.data.frame(t(dati), tree)<br>step2 <- step1[which(step1$cluster = n_cl), , drop = F] %>%<br>select(-c(cluster, n_col, features)) %>%<br>t()<br>cor_matrix_cl <- round(cor(step2, method = 'spearman'), 2)<br>return(cor_matrix_cl)}	threshold <- c(seq(0.5, 0.8, 0.05)) #thresholds choice between 0.5 and 0.8<br>ncluster <- c() #defined ncluster vector<br>for(k in threshold){<br>for (i in 1:150) #max number of clusters allowed 150 (arbitrary)<br>{tree <- data.frame(n_col = 1:dim(dati)[2], cluster = cutree(albero, k = i)) %>%<br>rownames_to_column("features")<br>minimum <- c() #defined the minimum vector<br>for (j in 1:i) {<br>cor_matrix_cl <- x(dati, tree, n_cl = j)<br>minimum <- c(minimum, min(abs(cor_matrix_cl))}<br>min_value <- sum(minimum > k) = length(minimum)<br>if (min_value = TRUE) break;}<br>ncluster <- c(ncluster, i)}<br>results <- cbind.data.frame(threshold, ncluster)

Table S7. Median values of radiomic features significantly different by molecular subtype after FDR correction at univariate analysis. IQR = interquartile range

Median Feature (IQR)	Molecular Subtype			
	**Basal-Like**	**Luminal A**	**Luminal B**	**Her2-Positive**
log-sigma-6-mm-3D_firstorder_Skewness	0.02 (−0.07–0.16)	−0.16 (−0.33–−0.01)	−0.09 (−0.20–0.004)	−0.18 (−0.48–−0.03)
lbp-2D_glrlm_ShortRunHighGrayLevelEmphasis	1.32 (1.31–1.34)	1.29 (1.27–1.31)	1.29 (1.28–1.31)	1.30 (1.29–1.31)
lbp-2D_glszm_LowGrayLevelZoneEmphasis	0.43 (0.42–0.45)	0.46 (0.44–0.48)	0.46 (0.44–0.50)	0.47 (0.45–v0.50)

Table S8. Radiomics Quality Score results. We obtained an intermediate score level of 19/36 (53%).

	**Criteria From Radiomics Quality Score**	**Points**
1	Image protocol of quality, well-documented image protocol	2/2
2	Multiple segmentation	1/1
3	Phantom study	0/1
4	Imaging at multiple time points	1/1
5	Features reduction	3/3
6	Multivariable analysis with non-radiomics features	1/1
7	Detect and discuss biological correlates	1/1
8	Cut-off analyses	1/1
9	Discrimination statistics and their statistical significance	2/2
10	Calibration statistic – resampling methods	2/2
11	Prospective study	0/7
12	Validation	0/5
13	Comparison to gold standard	2/2
14	Potential clinical utility	2/2
15	Cost-effectiveness analysis	0/1
16	Open science and data	1/4
	Total	19/36

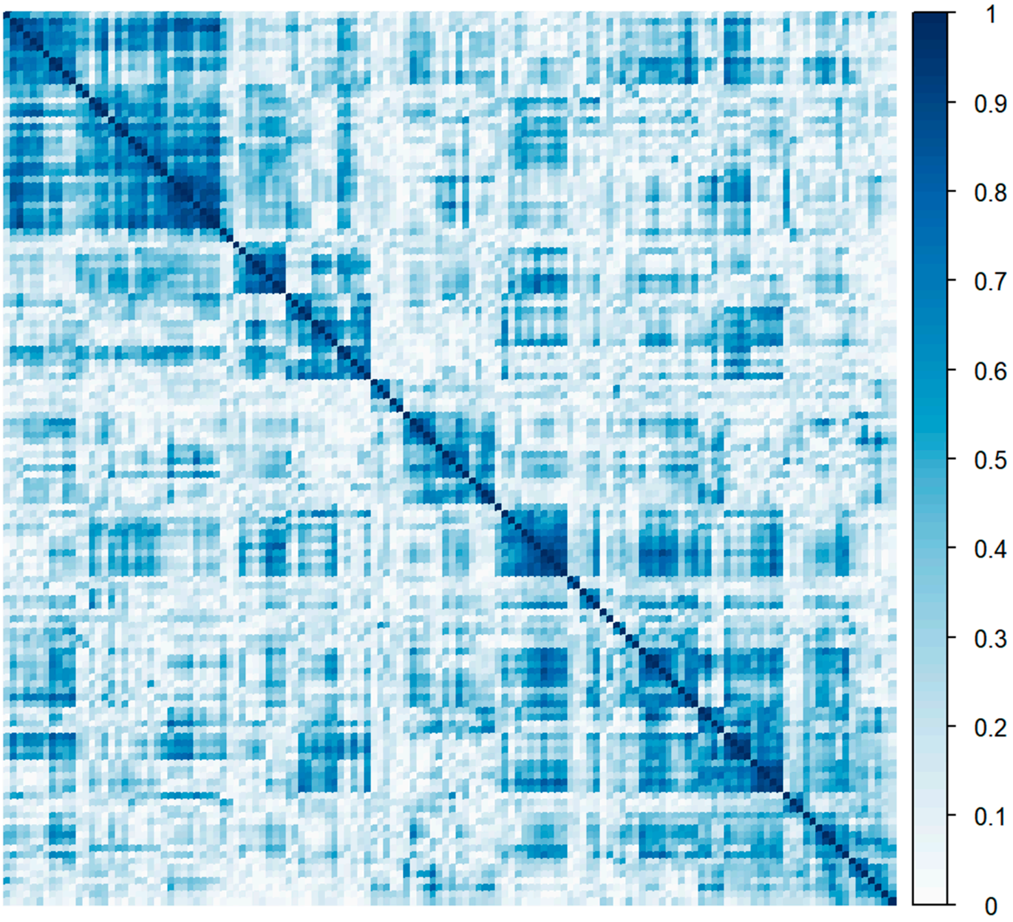

Figure S1. Graphical representation of the radiomic features clustering. The absolute value of the correlation coefficient (ranging from 0 to 1) between each pair of radiomic features is displayed, the 406 features being grouped into 136 clusters. The dark blue blocks along the diagonal indicate the high intra-cluster correlation.

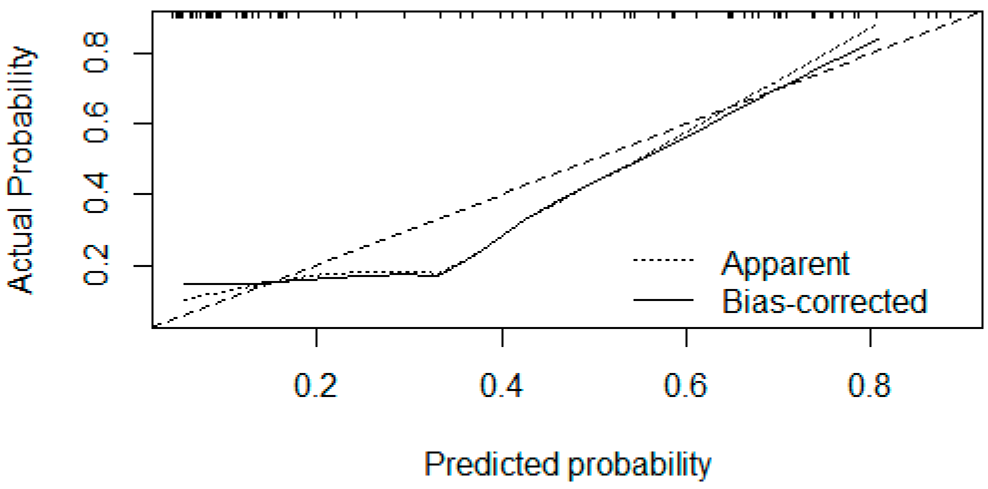

Figure S2. Calibration plot for the clinical/biological-radiomic model.
